# Reviewing the Great American Biotic Interchange: climate change as a trigger for biodiversity dispersal

**DOI:** 10.1098/rspb.2025.1745

**Published:** 2025-11-12

**Authors:** Roniel Freitas-Oliveira, Matheus S. Lima-Ribeiro, Victor H. Mendoza-Rodriguez, Levi Carina Terribile

**Affiliations:** ^1^ Programa de Pós-Graduação em Ecologia e Evolução, Instituto de Ciências Biológicas, Universidade Federal de Goiás, Goiânia, GO 74690-900, Brazil; ^2^ Instituto Nacional de Ciência e Tecnologia (INCT) em Ecologia, Evolução e Conservação da Biodiversidade, Goiânia, GO 74690-900, Brazil; ^3^ Instituto de Biociências, Universidade Federal de Jataí, Jataí, GO 75801-615, Brazil

**Keywords:** asymmetry hypothesis, cooling, GABI, neotropical region, biodiversity dispersion

## Abstract

The Great American Biotic Interchange (GABI), during which an intense biodiversity interchange occurred between South and North America (SA and NA), strongly affected the biodiversity of the Americas. Despite its importance, there are still knowledge gaps regarding the factors triggering species dispersion, the taxonomic groups that first dispersed, the age at which dispersions began and intensified, and whether there was a main dispersal direction through the continent (from NA to SA or *vice versa*). To fill these gaps, we conducted a scientific literature review of the GABI, searching for studies with information regarding dispersal age, taxonomic groups (invertebrates, amphibians, non-avian reptiles, birds, mammals and plants), dispersion direction (towards SA or NA) and the type of data used as the source of evidence (fossil, molecular or extant species). We also investigated the effect of the climatic dynamic on the biodiversity dispersal through the relationships between oxygen-isotope levels (δ^18^O, as a proxy of past temperatures) and the number and geological age of dispersal records. Only 41.8% (87 publications) of the studies included information on biodiversity dispersion during GABI. We found evidence of GABI starting at 23 million years ago (Ma) and becoming a continuous process from approximately 15 Ma. Cooling periods after the Miocene Climate Optimum favoured continuous dispersals, which have since intensified. Studies based on molecular data recovered more closely related to the intermediate ages of dispersal records. In addition, birds, plants and mammals were displaced first, whereas amphibians were displaced last.

## 1. Introduction

**Figure 1.**
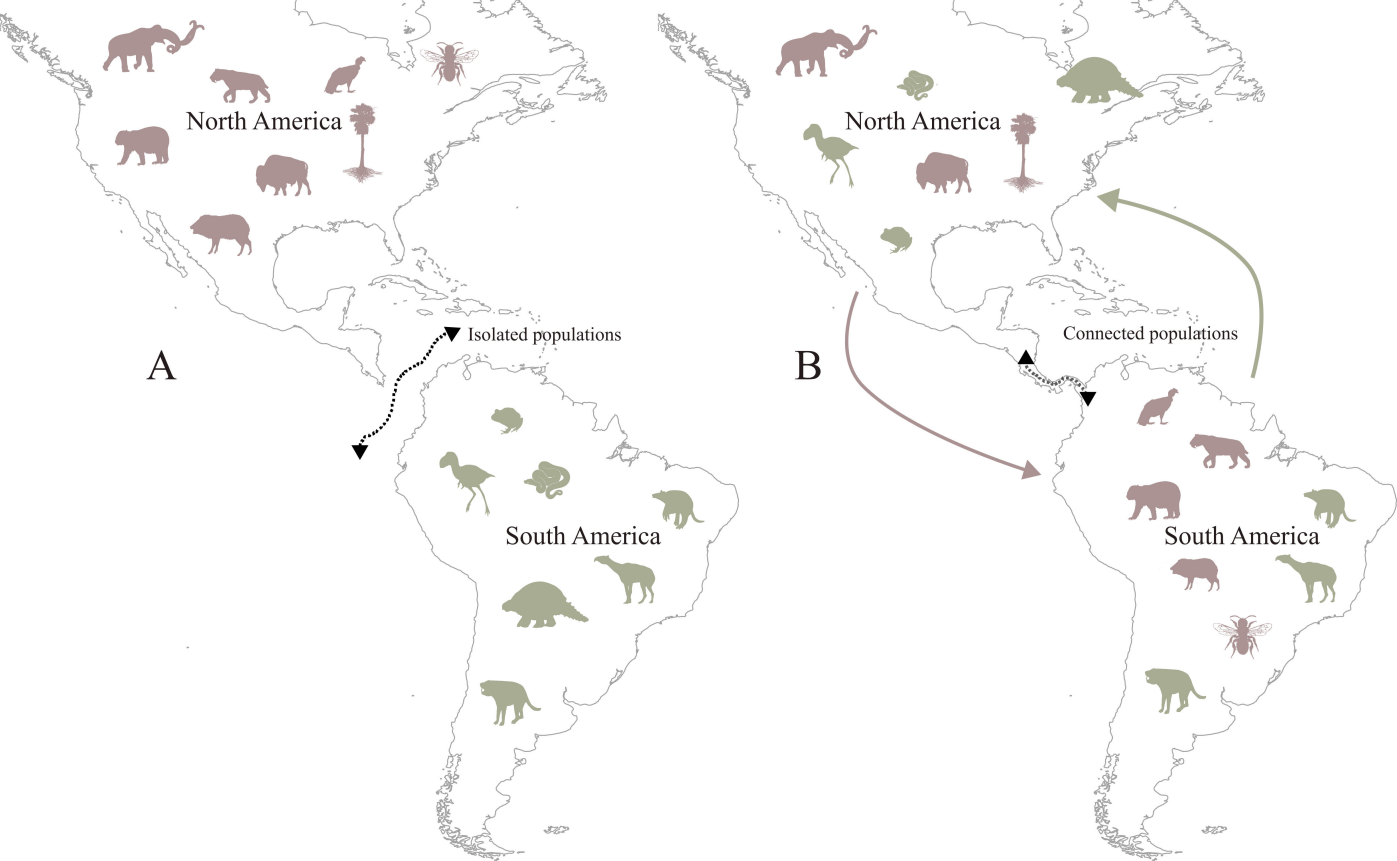
(A) Geographical context of North and South America isolation—indicated by dashed line—with representative fauna that evolved in isolation during this period; and (B) the Great American Biotic Interchange, denoting the end of the gap following the establishment of the Panama Isthmus land bridge—dashed line—and the faunal dispersion between North and South America. Brown silhouettes represent taxa from North America, while green silhouettes represent those from South America. The taxa silhouettes were obtained from PhyloPic (https://www.phylopic.org/): *Mammuthus columbi* by U.S. National Park Service (vectorized by William Gearty); *Titanis walleri* by Steven Traver; *Glyptodon* by Celest Luna; *Washingtonia robusta* by Guillaume Dera; *Boa constrictor* by Becky Barnes; *Bison* by Tracy A. Heath; *Craugastos augusti* by Jose Carlos Arena-Monroy; *Smilodon* by Margot Michaud; Megachilidae by Thomas Hegna; *Priodontes maximus* by Celeste luna and uploaded by Fabio Machado; *Tremarctos ornatus* by Andy Wilson; *Macrauchenia patachonica* by Steven Traver; *Vultur gryphus* by Kai Caspar; *Dicotyles tajacu* by Steven Traver; *Arminiheringia auceta* by Steven Traver. All silhouettes are under the Creative Commons CC0 1.0, Universal Public Domain Dedication license (https://creativecommons.org/publicdomain/zero/1.0/).

The Great American Biotic Interchange (GABI) is an important biogeographical event that occurred at the end of the Cenozoic, during which animals and plants that evolved in isolation dispersed from North America (NA) to South America (SA) and *vice versa* [1] (figure 1). The dynamics of the GABI have changed the biotic composition and shaped the current biodiversity of the American continent, including other processes, such as species diversification (e.g. beetles [2]), extinctions [3] and modification of biogeographical patterns (e.g. tropical avifauna [4]), and even inducing changes in species niches [5]. Historically, the closure of the Panama isthmus around 3 million years ago (Ma), a complex geological process involving fluvial connections, arc collisions and volcanism [6–8], has been considered a decisive event that made the GABI possible [9,10]. However, evidence of dispersion preceding the completion of the Panama land bridge [11], as well as the potential effects of environmental factors triggering species interchange [12], have challenged this conventional view of the GABI.

Although the closure of the isthmus has been related to an intensification in GABI dispersions at the end of the Cenozoic (e.g. GABI I, [13]), some studies claim that climatic fluctuations during that era may have been the precursor cause of most processes related to the GABI [12,14]. For example, changes in vegetation from tropical forests to savannah-like formations in Central America during the Quaternary ice age could have driven migration from the north to the south of the continent [14]. In addition, glacial conditions that preceded the GABI during the Late Miocene may have triggered species interchange [13,15]. Furthermore, periods of warming temperatures, such as the Middle Miocene Climatic Optimum (also called Middle Miocene Thermal Maximum, *ca* 16.9–14.7 Ma [16]), may have inhibited older dispersal or even have caused extinction events during the GABI [12]. However, the role of climate change remains unclear.

Studies on taxonomic groups that first dispersed have also been inconclusive. Molecular studies have supported the idea that plants dispersed before animals [11]. However, discrepancies in the proposed dispersal age for each group may be related to differences in the nature of the evidence (i.e. molecular data, fossil data or extant species) used to analyse the dispersion records. For example, based on molecular studies, the divergence of most animal groups occurred around 10–20 Ma [11], whereas based on fossil analysis [17], the first mammalian dispersion occurred at 8 Ma, which would have marked the initial stages of biotic exchange between NA and SA. Divergent ages resulting from these different sources of evidence add further uncertainties in the reconstruction of the time and dynamics of taxonomic dispersal during the GABI.

Another issue under debate is the asymmetry in the GABI. The asymmetry hypothesis considers that most mammals disperse from NA to SA, rather than *vice versa* [15,17,18]. The asymmetry hypothesis has already been tested and refuted for mammals, whose difference in the proportion of NA species found in SA is due to the high extinction rates of SA mammalian species [19]. However, the GABI was a multi-taxon dispersion event that included several groups of amphibians, birds, invertebrates, mammals, plants and reptiles [4,11,20], for which the asymmetry hypothesis of dispersal direction has never been formally tested in this wide taxonomic view.

We conducted a systematic review of the scientific literature to address the knowledge gaps about the GABI. Specifically, we investigated whether: (i) climate change triggered species dispersion across the Panama region; (ii) differences exist in the dispersion age related to the source of evidence (e.g. molecular, fossil or extant species); (iii) differences exist in the dispersal ages for different taxonomic groups involved in the GABI; and (iv) there were differences in the direction of dispersion, from NA to SA and *vice versa*.

## 2. Methods

We searched for information regarding the GABI in two online databases, Web of Science and Scopus, without language restriction, using a combination of the following keywords: ‘Great American Interchange’, ‘American Biotic Interchange’, ‘Great American Biotic Interchange’, ‘GABI’, ‘American Faunal Interchange’ and ‘Great American Faunal Interchange’. We performed a literature search from 2024 to the earliest available publications. We then confirmed that the results contained the keywords used in the search. We used the ‘stats’ package in the R environment [21] to remove the duplicate entries between the two databases by matching DOIs.

We used only peer-reviewed articles from scientific databases to extract the necessary information for our analyses (i.e. we did not consider grey literature such as reports, theses and meetings). From the selected studies, we recorded the target groups (amphibians, birds, invertebrates, mammals, plants and reptiles) and identified dispersing organisms at the family level. Additionally, we gathered information on the age of dispersion, the source of evidence used for dating the dispersal age and the dispersion direction. The dispersal age was obtained from the studies as follows. For each record of a given taxonomic group that participated in the GABI, we registered the age of that record and its continent of origin. For instance, a fossil record from SA dated to 5 Ma and whose continent of origin was NA had its dispersal age set to 5 Ma from NA to SA. We registered similarly records from molecular data or extant species. The dispersal age of a taxon was based on the data provided in the studies for a given dispersal, speciation or irradiation event related to the GABI, taking into account the continent of origin of the taxon. In addition, when information about the age of dispersion referred to a geological period (i.e. Miocene) instead of a specific date, we used the international stratigraphic chart (https://stratigraphy.org/chart) to obtain the age in Ma. When the record contained multiple dates or when the information was presented as a period range (e.g. Miocene, ranging from 23.03 to 5.33 Ma; thus, we use these two ages as records), we used the minimum and maximum age values. We also noted the source of evidence used to date dispersal age, recording if the study was based on fossil data, molecular data or extant species. Finally, for the dispersion direction, we noted whether the study was referring to dispersal from NA to SA and/or from SA to NA, respectively, or bidirectional. All data analyses were conducted using the computational environment R [21]. The figures were generated using the ‘ggplot2’ package [22].

### (a) Data analysis

To evaluate the role of the climatic dynamic in the dispersal, we used linear regression to search for relationships between: (i) geological age (Ma) versus oxygen-isotope levels (δ^18^O), (ii) geological age versus number of dispersal events, and (iii) δ^18^O versus number of dispersal events. The δ^18^O was represented as a mean for each million years [23]. Values of δ^18^O commonly used as a proxy for past temperatures were used here to investigate whether climate change may have driven biodiversity dispersal in the GABI. Because both δ^18^O and the number of dispersal events change through time, we used segmented regression to search for breakpoints at which the linear relationship changed, indicated by different slopes in the interaction. We fitted regression models with zero (i.e. simple regression), one, two or three breakpoints (models with four or more breakpoints were not possible owing to data limitations; see details below), and the optimal models were selected based on the corrected Akaike Information Criterion for small sample (models with ΔAIC <4). We used the ‘segmented’ function of the ‘segmented’ package [24] to search for breaking points (thresholds) along the interactions, and the ‘AICc’ function of the ‘MuMIn’ package [25] to select the optimal models. The ‘segmented’ function searches for breakpoints along the covariates that minimize the log likelihood of the relationships (i.e. the breakpoints that improve the adjustment of the relationships). The number of breakpoints is limited when a given interval in the covariates remains with a few observations (two or fewer observations by default), which prevents the estimation of the standard deviation for model parameters, including slope and breakpoints. Often, a few observations remain when breakpoints are too close to each other or at the boundaries. In our dataset, segmented regressions were possible with a maximum of three breakpoints.

Finally, principal component analysis (PCA) was performed to assess the relationships between the number of dispersal events and the type of evidence source, age of dispersion, taxonomic groups and dispersal direction. To compute PCA, we used the ‘prcomp’ function from the R base ‘stats’ to conduct all analyses [21].

## 3. Results

A total of 283 studies were retrieved from the search; however, only 208 were freely accessible. Of these, 87 studies provided information about dispersal during the GABI. We identified 325 dispersal records, corresponding to 95 families, with 245 records for mammals (40 families), 55 for birds (40 families), 9 for invertebrates (5 families and one phylum not considered in our analyses), 5 for plants (four families), 3 for non-avian reptiles (3 families), 1 for amphibians (1 family) and 1 for protozoan (1 family: Plasmodiidae) (see electronic supplementary material, table S1). Additionally, 285 records presented the source of evidence for determining dispersal age, and 302 records presented information on dispersal direction.

**Figure 2.**
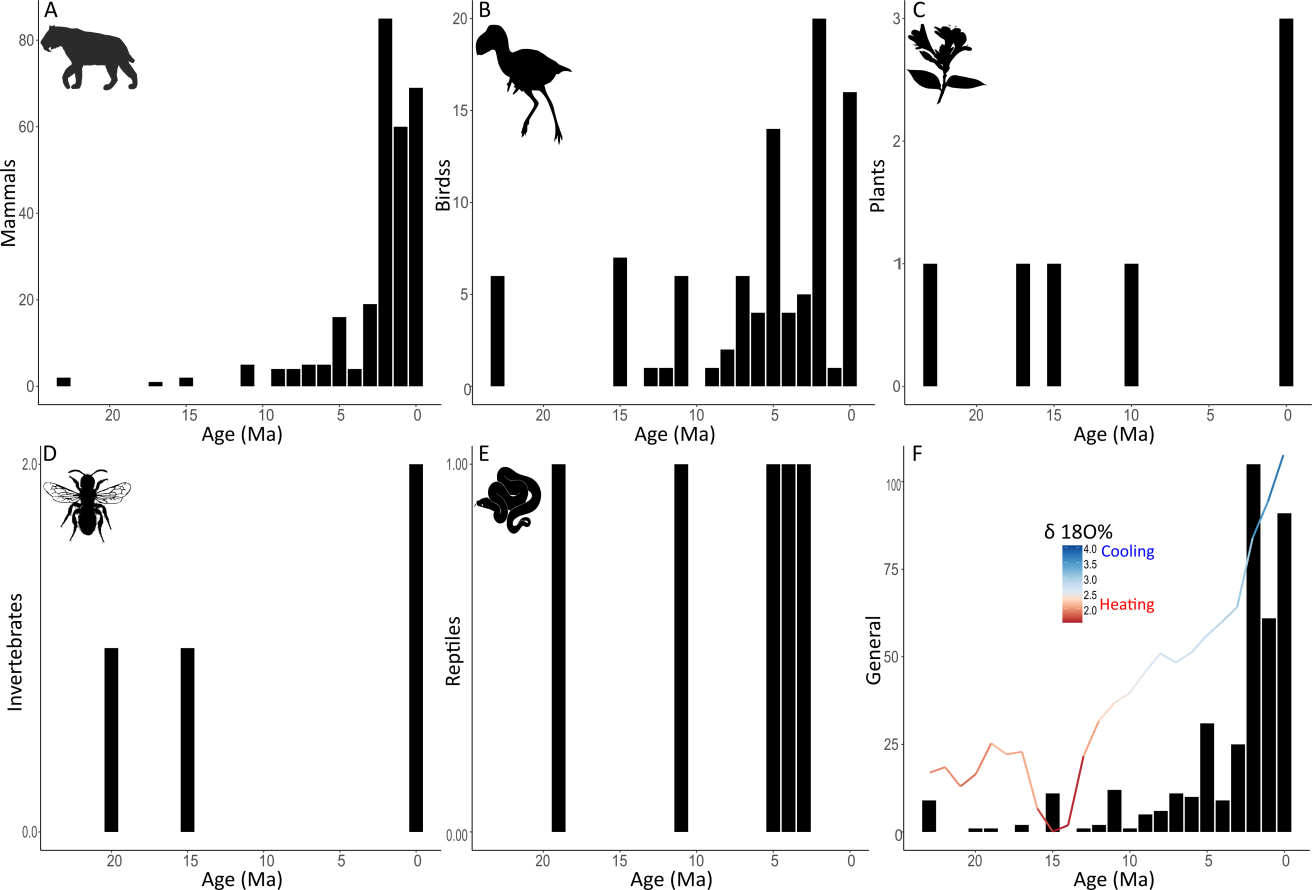
Number of dispersal events through the last 23 Myr of the GABI for (A) mammals, (B) birds, (C) plants, (D) invertebrates, (E) reptiles, and (F) general (all groups). The δ^18^O levels over time shown in (F) indicate heating (red) or cooling (blue) periods. The taxon silhouettes were obtained from PhyloPic (https://www.phylopic.org/): *Smilodon* by Margot Michaud; *Titanis walleri* by Steven Traver; Acanthaceae by Saneesh; Megachilidae by Thomas Hegna; *Boa constrictor* by Becky Barnes. All silhouettes are under the Creative Commons CC0 1.0, Universal Public Domain Dedication license (https://creativecommons.org/publicdomain/zero/1.0/).

**Figure 3.**
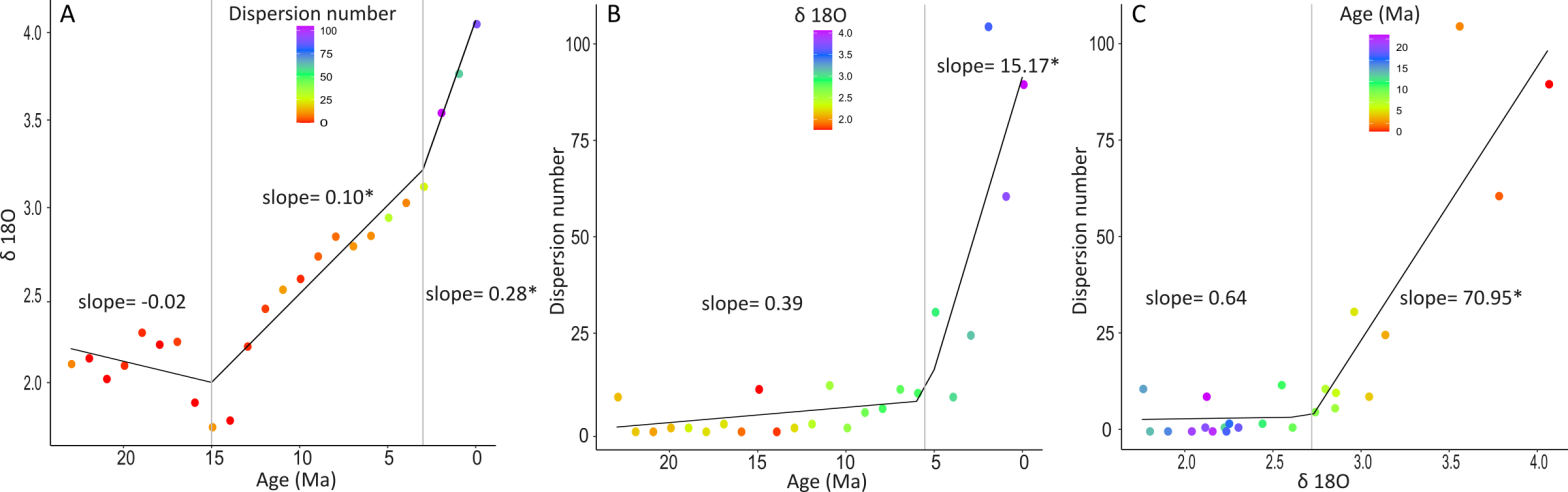
Segmented regression analysis showing the breaks (thresholds) for (A) δ^18^O levels over time (colours indicate the frequency of the dispersal events), (B) number of dispersal events over time (colours indicate the δ^18^O levels), and (C) number of dispersal events versus δ^18^O levels (colours indicate the age). The grey vertical lines represent the thresholds, and the * indicates the selected model (i.e. model with ΔAIC<4).

Overall, most included studies suggest that dispersal has been a continuous process, which became stronger from 15 Ma to the present (for a general view, see figure 2). We found two breakpoints of δ^18^O levels through time, one at 15 Ma and another at approximately 3 Ma (figure 3A). The levels of δ^18^O slightly decreased from 23 to 15 Ma (slope = −0.02) (warming conditions), and significantly increased since then (slope = 0.10) (cooling conditions), especially from approximately 3 Ma to the present (slope = 0.28; figure 3A). The frequency of dispersion tended to increase over time, especially from 5.5 Ma to the present (slope = 15.17; figure 3B). Furthermore, the dispersion events were almost constant at low levels of δ^18^O and remarkably increased at higher δ^18^O levels (slope = 70.95; figure 3C). In summary, the GABI dispersals began sporadically at 23 Ma and became significantly more frequent after 15 Ma, in close association with rising δ^18^O levels, especially in the last 3–5 Ma (figures 2F and 3).

**Figure 4.**
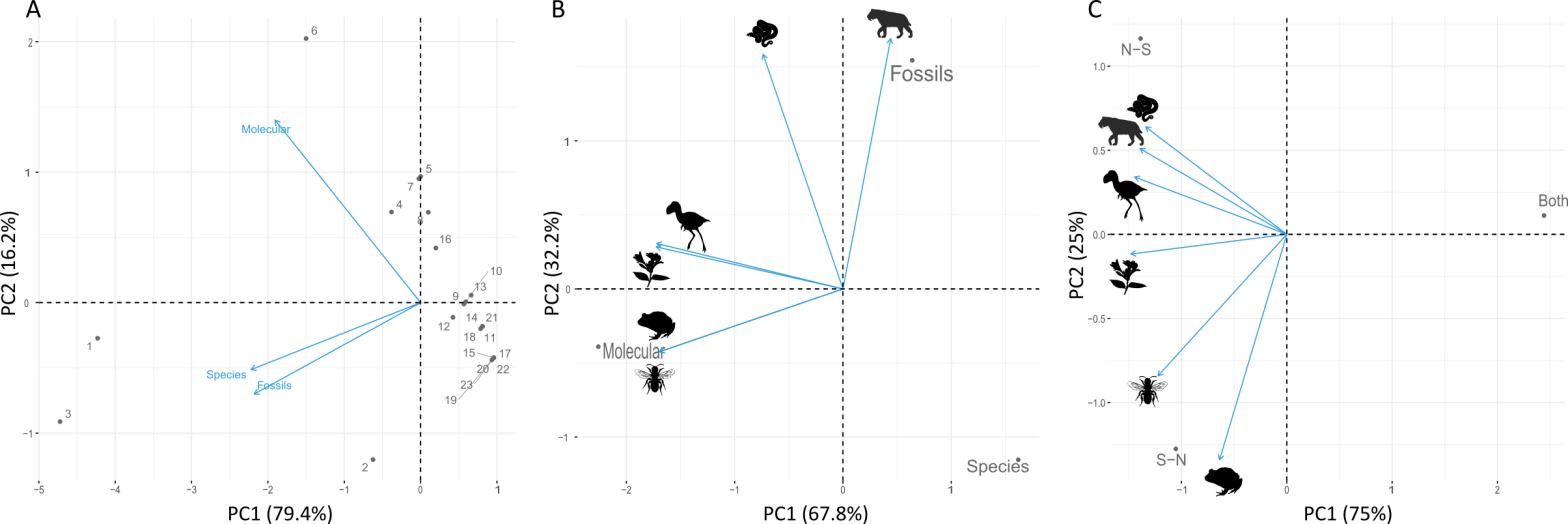
Principal component analysis relating the number of dispersal events with: (A) ages (Ma) and type of evidence source (molecular, fossil and extant species); (B) type of evidence source and taxonomic group (amphibians, birds, invertebrates, mammals, plants and reptiles); and (C) taxonomic group and dispersal directions (NA to SA, SA to NA, or both). The taxa silhouettes were obtained from PhyloPic (https://www.phylopic.org/): *Smilodon* by Margot Michaud; *Titanis walleri* by Steven Traver; Acanthaceae by Saneesh; Megachilidae by Thomas Hegna; *Boa constrictor* by Becky Barnes; *Craugastos augusti* by Jose Carlos Arena-Monroy. All silhouettes are under the Creative Commons CC0 1.0, Universal Public Domain Dedication license (https://creativecommons.org/publicdomain/zero/1.0/).

PCA analysis indicated that molecular evidence was related to dispersal records with intermediate ages (10–4 Ma), whereas the evidence based on fossils and extant species was related to both the most recent (<3 Ma) and oldest dispersals (>20 Ma; see figure 4A). In addition, the molecular evidence was mostly related to invertebrates, amphibians, plants and birds, whereas the fossil evidence was related to mammals and reptiles (figure 4B). Regarding to the dispersal age of the taxonomic groups, the oldest records were from mammals, birds (from 23.03 to 0 Ma; figures 2A,B and 4A) and plants (from 23.03 to 0.011 Ma; figure 2C), suggesting they were the first ones to participate in the GABI, followed by invertebrates (20.5–0.3 Ma; figure 2D) and reptiles (19.3–3.4 Ma; figure 2E), and subsequently amphibians and protozoans (both with records at 12 and 6 Ma). Furthermore, amphibians, invertebrates, plants and reptiles presented dispersal pulses that were more widely spaced over time than for birds and mammals (figure 2). Finally, the taxonomic groups did not present clear preferences in the direction of dispersal (N–S, S–N, or both), except for amphibians, which dispersed predominantly from SA to NA (figure 4C). The first two axes of all PCAs explained more than 80% of the variation among the variables (figure 4).

## 4. Discussion

In this study, we systematically compiled and analysed literature on the GABI to elucidate the influence of climate dynamics, differences in dispersal ages by evidence type and taxonomic group, and asymmetry in dispersal direction. Our findings indicate that the GABI began long before the closure of the Isthmus of Panama, as revealed by fossil evidence, and intensified over time in close association with climate dynamics. The frequency of dispersal increased continuously with increasing δ^18^O levels (figures 2F and 3C). The rise of δ^18^O levels indicates the onset of the cooling phase, at approximately 15 Ma (figure 3A), shortly after the Middle Miocene Climatic Optimum (a warm period spanning *ca* 16.9–14.7 Ma), at which point the GABI becomes a continuous event of dispersions between the Americas (figure 2F). The even more pronounced cooling climates, evidenced by increasing δ^18^O levels since 3 Ma, further intensified the dispersions. Notably, the formation of the land bridge across the Isthmus of Panama progressively facilitated these dispersals, especially for taxa with lower dispersal capacities, such as reptiles and amphibians. This evidence strongly suggests that past climate dynamics triggered dispersal across the GABI [12].

### (a) Effect of climate change on the Great American Biotic Interchange

The low δ^18^O values before 15 Ma indicate a warm period when the GABI was not continuous, with only sporadic dispersal events (figure 3A). Warm conditions have been widely described as a limiting factor for biodiversity [26]. The thermal adaptation theory argues that it is more challenging for biodiversity to adapt to heating than to cooling climates [26]. Consequently, most terrestrial species from temperate regions present a lower occupation than expected in warm areas that are within their warmer thermal tolerance [27]. In the context of the GABI, mammalian extinction in SA was associated with warming phases, whereas dispersal events were positively affected by cooling periods [12]. Therefore, the warmer temperatures preceding and during the Middle Miocene Climatic Optimum, as indicated by lower δ^18^O values before 15 Ma (see also [28]), may have acted as a significant barrier preventing biodiversity dispersal during the initial stages of the GABI.

In contrast, the increase in δ^18^O levels between 15 and 3 Ma (figure 3A) indicates a cooling period associated with an increase in dispersal events (see blue and purple in figure 3A,B), a period in which the GABI became a continuous event (figure 2F). This period began after the temperature drop that occurred at the end of the Middle Miocene Climatic Optimum, as indicated by increased δ^18^O levels (figure 3A). This may have reduced the barrier to dispersion formed by an increase in temperature in the past. The role of cooling in GABI dispersions has already been described in the literature. Previous studies in recent decades have suggested that cooling, ice age, or climate change may have affected species dispersal during the GABI [13–15]. However, this hypothesis has only been tested in mammals, showing that cooling periods increased mammal dispersion rates between the Americas during the last 5 Myr [12]. Here, we corroborate these findings with several documented dispersal events for various taxonomic groups (amphibians, birds, invertebrates, plants and reptiles) and over a longer time span (from 23 Ma to the present).

Our findings suggest that the cooling period was responsible not only for making GABI a continuous event but also for intensifying dispersion more recently. Between 3 and 0 Ma, there was a remarkable increase in δ^18^O levels, indicating a period with even greater cooling, which could have facilitated dispersion events (figures 2F and 3A; see below). This dispersion may have been further strengthened by the formation of the Isthmus of Panama around 2.6 Ma and is referred to as GABI I ([13]; see figure 2F). The intensive drop in temperature (indicated by increased δ^18^O levels; see blue and purple colours in figure 3B), along with the formation of the land bridge, likely created favourable conditions that accelerated biodiversity interchange between the continents.

Once cooling enabled species dispersal in the GABI, species that reached a new environment may have settled readily during cooling periods, as they were more likely to adapt to cooling than to warming conditions [26]. This was likely true for species from temperate regions, which were better able to occupy their expected cooling limits [27]. Moreover, tolerance to cold environments evolves faster than tolerance to warm environments [26,27,29,30]. Species from temperate zones often struggle to occupy their warmer marginal niche well enough to avoid competitive interaction with stronger competitors; this difficulty increases with latitude [27]. However, the role of biotic interactions in the GABI, particularly during warmer periods, remains debated.

### (b) Literature trends

In this study, we unveiled that only 41.8% of freely available publications included information regarding biodiversity dispersion during the GABI. This suggests that the GABI was not the primary focus of most studies (approximately 58%) and was only cited, but not particularly investigated—for example, a reference to the GABI to strengthen a discussion about *Equus* dispersion (see [31]). Indeed, although we found more than 200 studies using the term GABI, most did not address specific issues regarding this event. Thus, the GABI remains an open field for investigation. Recent advances in methodological approaches based on ecological niche modelling and the increasing availability of palaeoclimate data (e.g. palaeoclimate of 5–0 Ma, PALEO-PGEM v. 1.0 [32]) have paved the way for further studies to elucidate dispersal movement along the American continent, especially for the 95 families we recorded here.

Our results partially agree with those of previous studies, indicating that plants were the first to disperse [11,33], although we also found that mammals and birds dispersed during the same period. It is plausible that these three groups were the pioneers, given their superior dispersion abilities: the wind dispersed seeds, the flying ability of birds and the high dispersal capacity of mammals, mainly due to their body masses (e.g. *Panthera onca*, a large mammal that can travel an average distance of 18.3 km per day, [34]). Plant dispersal is facilitated by mammals and birds (e.g. megafauna could disperse plant seeds over 6 km [35]). In contrast, invertebrates dispersed after these three groups, particularly those with flying abilities, and parasites that might have dispersed through their hosts. That the latest groups to disperse were reptiles and amphibians is justified by their low dispersion ability and smaller body sizes: for example, the lizard *Liolaemus quilmes*, with an estimated home range of 42–350 m² [36], the frog *Ameerega trivittata*, with a home range of 177.93 ± 229.17 m² [37], and other water-dependent amphibians.

Studies based on molecular evidence have suggested intermediate ages for the GABI [11]. Molecular analyses tend to capture the divergence signal in the exact period in which it occurs (e.g. at phylogenetic nodes), which differs from fossil data [38]. Fossilization is a complex and non-uniform process subject to ecological traits (e.g. population abundance) and taphonomic biases (e.g. fossil weathering: chemical or mechanical destruction over time, [39]). Therefore, we hypothesize that the older dispersions are recorded in fossil data but not in molecular approaches because molecular analyses capture lineage divergence, and the older dispersions may not result in speciation. Consequently, the intermediate dispersions inferred from molecular data may represent the stage at which the GABI promoted lineage adaptation and speciation. However, this hypothesis needs to be tested in future studies.

We also found a relationship between the evidence sources (molecular, fossil or extant species) and some taxonomic groups studied in the GABI literature. Birds, plants and mammals were the most extensively studied groups, followed by reptiles and amphibians, whereas all invertebrates (e.g. Polychaeta, Gastropoda and Insecta) were significantly underestimated based on global occurrence data [40]. This bias in our data, with abundant mammal and bird records, probably existed because these groups are more charismatic than the others (reptiles, amphibians, invertebrates and plants) and because of the greater success in the fossilization of mammals and birds. A more thorough evaluation, including underrepresented taxa, is necessary to gain comprehensive insights into biodiversity trends throughout the GABI.

We found no concrete evidence of a difference in dispersal direction (SA to NA or *vice versa*) between the groups (birds, invertebrates, mammals, plants and reptiles). This reinforces the idea of similar dispersion along the American continent for most groups (except for amphibians), as previously observed for mammals (see [19]), and undermines the hypothesis of asymmetry in the biodiversity interchange. However, we did not find a pattern of dispersal direction for amphibians, which needs to be interpreted with caution and requires further investigation because of the lower number of dispersion records for this group.

In conclusion, this study advances current knowledge of the GABI through a comprehensive review. By integrating temporal analyses of dispersal patterns, evidential records, and oxygen isotope levels, we demonstrate that although this event began in the Early Miocene, and became continuous from around 15 Ma, coinciding with a cooling phase. Furthermore, molecular evidence aligns more closely with intermediate ages for dispersal in comparison with the ages indicated by fossil records or extant species. Finally, it is possible that the dispersal abilities of mammals and birds facilitated their early dispersal through the American continent.

## Data Availability

The data are available in the supplementary material and in the Dryad repository [41]. Supplementary material is available online [42].
